# Children passively allow other’s rule violations in cooperative situations

**DOI:** 10.1038/s41598-018-25210-4

**Published:** 2018-05-01

**Authors:** Ayaka Ikeda, Yuko Okumura, Tessei Kobayashi, Shoji Itakura

**Affiliations:** 1 Department of Psychology, Graduate School of Letters, Kyoto University, Kyoto, Japan; 2 NTT Communication Science Laboratories, Kyoto, Japan

## Abstract

Recent studies in developmental psychology have revealed the developmental origins of cooperation. Although such studies regard cooperation as a pro-social behavior, studies on adults have found a negative aspect: cooperation sometimes promotes unethical behavior. Adults also exhibit altruistic cheating, even though their cheating might not actually benefit them. However, the development of negative aspects of cooperation remains unclear. Our study examined whether 7-year-old children engage in negative aspects of cooperation from two aspects using a peeking paradigm. Specifically, Experiment 1 examined children’s negative aspects of cooperation from the perspective of collaboration and Experiment 2 examined altruistic behavior. Results of Experiment 1 revealed that children kept the cheating of a collaborative partner secret even though they did not actively cheat themselves. In Experiment 2, children also kept the partner’s cheating secret even when violations did not provide any reward to themselves, if the predefined reward was high. In contrast, children did not keep the cheating secret if the predefined reward was low. Overall, our findings suggest that even 7-year-olds tend to act as if cooperating is more important than following rules that are compatible and exhibit negative aspects of cooperation.

## Introduction

Cooperation is essential for maintaining social order. Many studies have focused on the positive aspects of cooperation, such as achieving group goals, helping, sharing resources, and informing^1–6^. However, studies in behavioral economics have shed light on the negative aspects of cooperation, suggesting that adults violate rules or cheat more often in collaborative situations than when alone^7^. Adults also have been found to behave unethically in ways that profit others without benefitting themselves^7–9^. However, to date, the development of negative aspects of cooperation remains unclear.

The negative aspects of cooperation are thought to derive from the dilemma between rule violations and cooperation. In the field of developmental psychology, the development of cooperation and rule violations has been studied independently. Cooperation has been studied by dividing it into the development of altruistic behavior and collaboration. Whereas altruistic behavior is an action in which an individual sacrifices for another, multiple individuals work together for mutual benefit during collaboration^10^. Many researchers have observed altruistic behaviors, such as helping, sharing, and informing in 1- to 2-year-olds^1–4^. In contrast, intentional collaboration, in which two or more people act to their shared goals, starts around 3 years of age^1,5,6^.

Regarding sensitivity to rule violations, children have been found to give normative protection to other’s rule violations at around 3 years of age^11^, and 6-year-olds mete out third-party punishments to a rule breaker, even though such rule violations do not affect their own risk-benefit^12^. As for rule breaking by children themselves, children around the age of 3 start lying about their own rule violations^13^. Such falsehoods become more sophisticated as they grow older, and 7-year-olds can weave coherent stories that camouflage their lies^13^. In addition, children around 5 years of age start to care about the existence of others and their evaluations of them, and they are less likely to violate the rules when they are seen as opposed to when they are not seen^14^. As shown above, the awareness of rule violations starts to gradually develop simultaneously with the development of collaboration.

While many findings have concluded that children show an awareness of cooperation and rule violations, few studies have examined how children behave when cooperation leads to rule violations. One of the few studies that examined children’s responses to the dilemma between cooperation and rule violations is a study by Gräfenhain, Carpenter and Tomasello^15^, that examined how 3-year-olds handled a rule violation by an adult while completing a collaborative task or non-collaborative task^15^. They found that the frequency of the occurrence of tattling behavior did not change regardless of whether the adult was a task partner or not. This finding suggests that 3-year-olds do not yet exhibit negative aspects of cooperation.

“Blue lies” (i.e., are lies for collective merit) are another example of the dilemma between cooperation and rule violations. Fu, Evans, Wang, and Lee^16^ examined the development of blue lies in elementary school children (7-, 9-, and 11-year-olds)^16^. In this study, children were asked to select players for a school chess championship from their class in accordance with selection rules. Next, an experimenter induced the children to violate the rules. On a subsequent day, the children were asked whether they had selected players by following the rules. The proportion of children who answered that they had followed the rules increased with age. Such lying to benefit the group suggests that children exhibit negative aspects of cooperation beginning from at least the age of 7.

Moreover, adults violate rules for their partner’s merit in collaborative tasks even without benefit to themselves^7^. This excessively altruistic behavior is sometimes called “altruistic cheating”^9^. Previous studies reported that altruistic cheating in adults may occur because violations are verified by profiting over other persons or groups^9,17,18^. Even though breaking rules is not considered good, these violations are perceived as lighter than in actuality or are rationalized since they are profiting another person or group. Thus, it seems that adults believe that providing profit to others is more important than maintaining rules. In this way, although altruistic cheating is also one behavioral component of negative cooperation, this aspect has not yet been examined in children.

There are two types of rule violations depending on how people engage in the negative aspects of cooperation, namely, actively breaking rules and passively breaking rules by keeping a collaborator’s rule breaking secret. Because the rule violation in Fu *et al*.^16^, was fueled by the experimenter, it is not clear which role children play. This viewpoint is important because clarifying the role of negative aspects of cooperation in childhood might elucidate the mechanisms underlying its development.

Our study examined the negative aspects of cooperation in 7-year-olds from two aspects: collaboration and altruistic behavior. First, we investigated whether children actively or passively break rules when rule violations increase rewards for both of the child and partner. This experiment was designed to clarify the ways in which children engage in negative aspects of collaboration. Second, we examined whether children exhibit altruistic cheating when rule violations only increase their partner’s rewards. In order to examine these issues, it was necessary to recruit children who engage in negative aspects of cooperation. Thus, we targeted 7-year-olds since previous studies have reported negative aspects of cooperation in this age group^16^, although the processes underlying this engagement are unclear.

We conducted two experiments to clarify the above issues. Experiment 1 examined the ways in which children engage in negative aspects of collaboration by comparing when a child and another adult player individually work on a task versus when they work collaboratively. Experiment 2 examined whether altruistic cheating occurred, and, if so, how children engage in this behavior by designing conditions in which the child’s performance only affected the partner’s reward and did not affect his or her own reward.

## Results

### Experiment 1

**Figure 1:**
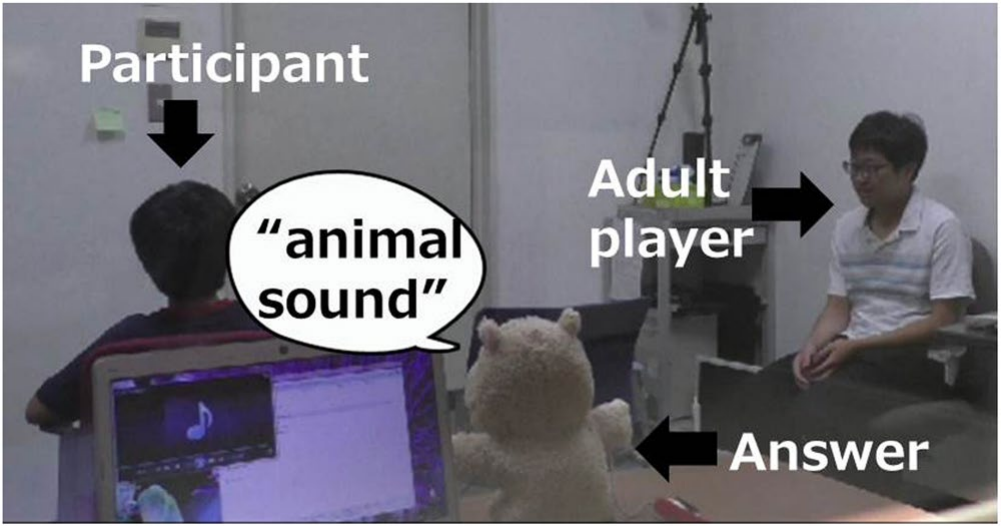
Study snapshot: Child is an answerer and an adult player is a non-answerer. The quiz answer was a puppet that covers a speaker. Experimenter sat behind the puppet and laptop PC.

Children were randomly divided into two conditions, namely, the individual condition or the collaborative condition. In both conditions, a child and another adult player earned coins based on their task performance and could exchange them for stickers. In the individual condition, a child and another adult player individually gathered coins and received rewards depending on the amount of earned coins. In the collaborative condition, a child and another adult player gathered coins as a team and shared the obtained rewards depending upon the number of coins. We used a modified version of a peeking paradigm^19^. In this modified design, two persons sequentially joined the quiz under a situation where they presented each other’s quiz; that is, the non-answerer knows the answers (Fig. 1). With this modification, we can distinguish whether participants are actively or passively cheating. If participants initiate cheating when they respond or provide hints to the other player when they are in the non-responder role, they are regarded as actively cheating. If they do not violate the rules by themselves and maintain another player’s violation secret, they are labeled as passively cheating. Tattling by participants on the rule violations of others was used as an index of passively cheating. We used this index since tattling is a behavior related to obeying rules and children over 4 years old tattle about rule violations by unfamiliar adults^20^.

**Figure 2:**
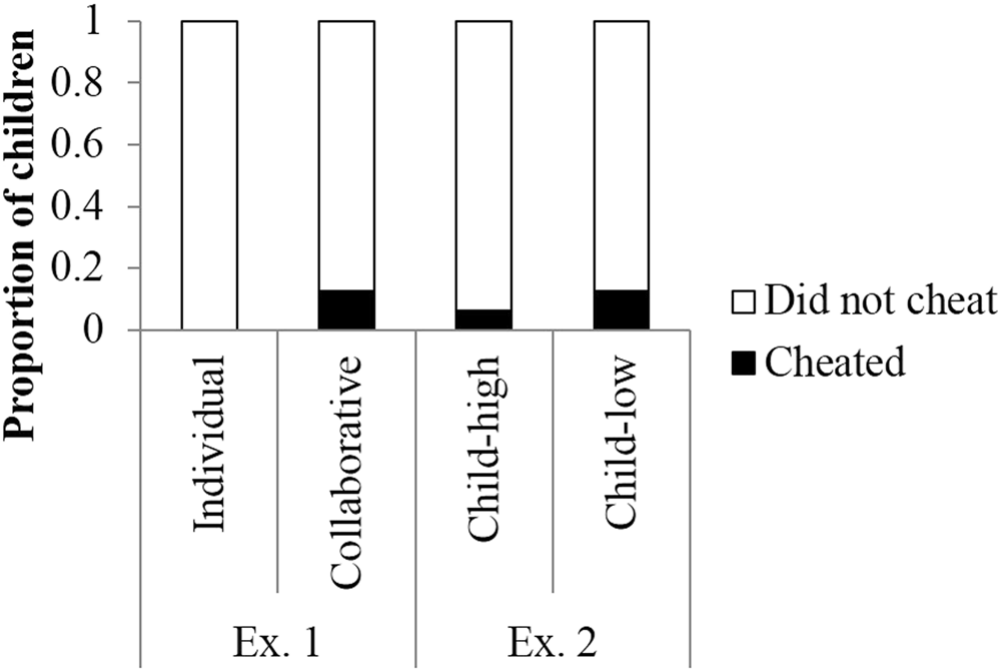
Proportion of children who did/didn’t cheat. Children who cheated were engaging in negative cooperation, which is an index of actively cheating.

**Figure 3:**
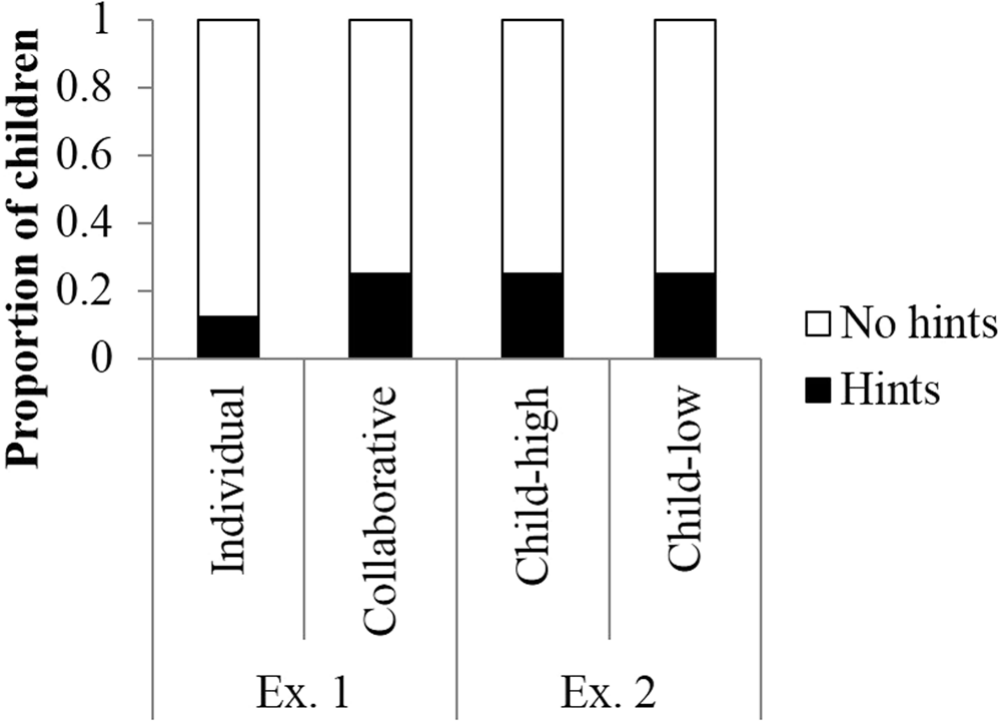
Proportion of children who provided hints to adult players. Children who provided hints were engaging in negative cooperation, which is an index of actively cheating.

**Figure 4:**
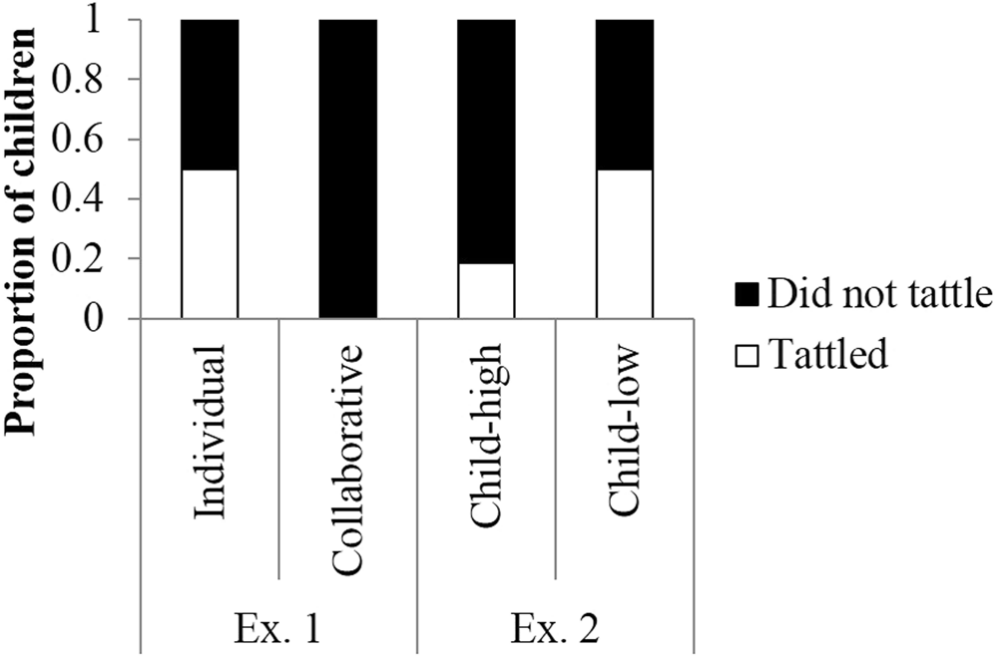
Proportion of children who tattled about player’s cheating. Children who did not tattle were engaging in negative cooperation, which is an index of passively cheating.

Figure 2 shows the proportion of children who cheated during the game. Binomial tests revealed that fewer children cheated during both conditions (individual condition: 0 of 16 children, *p* < 0.001; collaborative condition: 2 of 16 children, *p* = 0.004). Figure 3 shows the proportion of children who gave hints to the adult player. Fewer children provided hints in both conditions (individual condition: 1 of 16 children, *p* = 0.004; collaborative condition: 4 of 16 children, *p* = 0.07). The children rarely provided answers to the quiz, but they did provide hints, such as the characteristics and habits of the animal. Figure 4 shows the proportion of children who did not tattle on the adult players. Although we identified no difference between the number of children who tattled and those who did not report in the individual condition (8 of 16 children, *p* = 1.00), the number of children who did not tattle was significantly less than those who tattled in the collaborative condition (0 of 16 children, *p* < 0.001).

As a result of Fisher’s exact test, no differences between conditions were identified in terms of the proportion of children who cheated (*p* = 0.48) or the proportion of children who provided hints to the adult player (*p* = 0.65). However, there was a significant bias in terms of the proportion of children who tattled on the adult players (*p* = 0.002), such that children in the collaborative condition tattled less than children in the individual condition.

These findings suggest that we found one negative aspect of collaboration in 7-year-olds. Specifically, their engagement in the negative aspects of collaboration was accomplished by keeping the adult player’s cheating secret, rather than by cheating themselves or providing hints to the adult players. In other words, 7-year-olds passively cheated by keeping the adult player’s cheating secret without engaging in violating the rules themselves.

### Experiment 2

In Experiment 2, we examined whether altruistic cheating occurred when the child’s performance only affects the partner’s reward and the child’s reward is predefined. In such cases, the amount of the child’s reward is important because it might influence their behavior^21,22^. Thus, we created two conditions where children were predefined as receiving a high or low reward and examined whether the amount of the child’s reward affected their altruistic cheating. If children manifested altruistic cheating even though their rule breaking did not provide any merit to them, their behavior would resemble the collaborative condition results in Experiment 1. Moreover, we expected the children to show more negative aspects of cooperation when they received greater rewards relative to when they received lower rewards.

Children were randomly divided into two conditions, namely, the child-high condition or the child-low condition. In the child-high condition, a child and an adult player gathered coins as a team. While the adult player’s reward was determined by the sum of coins earned by the team, the child’s reward was predefined as six stickers regardless of the number of coins. In the child-low condition, although a child and an adult player earned coins as a team, the adult player’s reward was determined by the amount of coins gained by them together. The child’s reward was predefined as one sticker regardless of the number of coins. Except for a change in the amount of the child’s fixed reward, this procedure was identical to the collaborative condition in Experiment 1.

Binomial tests revealed that fewer children cheated in both conditions (child-high condition: 1 of 16 children, *p* = 0.0001; child-low condition: 2 of 16 children, *p* = 0.0041; Fig. 2), and fewer provided hints in both conditions (both conditions: 4 of 16 children, *ps* = 0.08; Fig. 3). In addition, although there were no differences between the number of children who tattled and those who did not in the child-low condition (8 of 16 children, *p* = 1.00). Significantly fewer children did not tattle relative to those who did tattle in the child-high condition (3 of 16 children, *p* = 0.02) (Fig. 4).

We combined the Experiment 1 results and compared the proportion of children who exhibited target behaviors between conditions. There were no differences between conditions in terms of the proportion of children who cheated (*p* = 0.74, Fisher’s exact test) or the proportion of children who provided hints to the adult player (*p* = 0.81, Fisher’s exact test). However, there was a significant bias in terms of the proportion of children who tattled on the adult player (*p* = 0.001, Fisher’s exact test). Multiple comparisons revealed that the children in the collaborative condition tattled less than the children in the individual and child-low conditions (*ps* = 0.007, Benjamini & Hochberg method).

Taken together, fewer children cheated by themselves or provided hints to the adult player, similar to the results of Experiment 1. In terms of tattling behavior, we identified no differences between the proportion of children who tattled on the adult player in comparison to those who did not tattle in the child-low condition, and this tendency was similar to the individual condition of Experiment 1. Although the proportion of children who tattled in the child-high condition did not differ from the other conditions, significantly more children did not tattle. These results suggest that 7-year-olds engaged in altruistic cheating by passive rule breaking when their reward was maintained at a high level.

## Discussion

This study investigated the extent to which 7-year-olds engage in the negative aspects of cooperation from two perspectives. Experiment 1 revealed that although 7-year-olds did not initiatively break rules, they passively violated rules by failing to tattle on adult players in a collaborative situation. Experiment 2 revealed that, although children tended to altruistically accept violations by their partner, their cooperation was limited when they received a high reward. These results suggest that cooperation is more important than conforming rules for children, although both cooperation and conforming to rules are also crucial.

In our experiment, children rarely actively cheated regardless of the differences between conditions. They did not actively break the rules, suggesting that they understood the rules and intended to follow them. Considering that a previous study reported that 70–90% of 6- to 7-year-olds cheated in the peeking paradigm when they participated alone^19^, the adult players in our study might function as an effective monitor for children. Our result is consistent with findings that cheating behavior in 5- and 8-year-olds decreased^14^ and 5-year-olds stole less when they were monitored by another person^23^. These prior findings suggest that the presence of others did not induce active violations by children, but inhibited them. On the other hand, the 7-year-olds in our study accepted violations by not tattling in collaborative situations. That is, children who collaborated with the adult partner tended to show more negative aspects of cooperation relative to those who individually participated in the quiz, suggesting that a child’s tendency to deliberate between cooperation and conforming to rules is fragile and likely influenced by their partnerships with others. The children in our study did not initiate violations but accepted them whenever the adult partner did, suggesting that cooperation in collaborative situations may be more important as opposed to following rules to them.

Since Gräfenhain, *et al*.^15^ revealed that the tattling behavior of 3-year-olds did not change depending on whether the child and the adult player were collaborating^15^, developmental changes from ages 3 to 6 might exist to explain negative aspects of cooperation in collaboration. For example, it is possible that the development of negative aspects of collaboration is influenced by the recognition of how group members behave. Plötner, Over, Carpenter & Tomasello^24^ reported that 5-year-olds thought that collaborative members should help each other^24^. Actually, 5-year-olds (but not 3.5-year-olds) chose to help collaborative partners rather than non-collaborators^25^. Thus, perhaps the children in the collaborative condition of the present study thought that they should help their partner and chose to keep their partner’s cheating secret. At the same time, the children’s recognition of in-group members also changed, such that 5-year-olds (but not 3.5-year-olds) chose to help in-group members rather than out-group members^25^. In addition, Jordan *et al*.^12^ found that 6-year-olds punished a dictator more in a dictator game when an out-group member decided to share unequally with an in-group member than the reverse^12^. In Experiment 1, when the child and the adult player collaboratively participated in the quiz, the adult player resembled an in-group member because they were on the same team. Conversely, when the child and the adult player participated in the quiz individually, the adult player resembled an out-group member because they were irrelevant. As a result, it is possible that children prioritize cooperation in the collaborative condition. This result could also be based upon reputation management, since even 5-year-olds are more concerned about their reputation as an in-group member than an out-group member^26^. Even though it is unclear how children regard adult players, recognition of how group members behave seems to affect children’s behavior.

The second possibility for the development of negative aspects of cooperation is the development of a child’s ability to calculate loss and gain. In third-party punishment, 6-year-olds, unlike 5-year-olds, can decide to give punishment based upon its cost^27^. When the child and the adult player are on the same team, the partner’s cheating profited the children in comparison to when they individually participated in the quiz. Thus, the ability to calculate loss and gain is critical for the negative aspects of cooperation.

When children participated in the quiz with their partner on the same team, they tended to avoid tattling even when cheating did not affect their reward, suggesting that 7-year-olds altruistically follow violations for their team member. However, this tendency is limited when rewards are kept at a high level. When a child’s reward was fixed at a lower level, their degree of cooperation with the partner resembled the degree of cooperation that occurred when the child and the adult player individually participated in quizzes. This result corresponds to the adult data in a prior study by Gino & Pierce^21^. According to Gino & Pierce^21^, this difference in behavior results from a modification in the degree of emotional distress caused by an inequality in the reward. Recent developmental studies have revealed that children are sensitive to inequality. Fehr, Bernhard & Rockenbach^28^ reported that 3- to 8-year-olds prefer equal distribution over unequal distribution, and this tendency intensifies with age^28^. Seven-year-olds start to care not only about unequal distribution, in which they are given smaller reward, but also unequal distribution, where they are given bigger rewards^22^. Thus, it is possible that the children tried to decrease the emotional distress caused by inequality by keeping their partner’s cheating secret when the reward exceeded their partner’s reward.

Furthermore, altruistic cheating in adults may occur because violations are verified by profiting over other persons or groups^9,17,18^. Even though breaking rules is not good, their violations may be perceived as being lighter than they actually are or are rationalized because another person or group is profiting. Although we did not confirm this possibility in our study, perhaps this psychological mechanism allows children to altruistically engage in the negative aspects of cooperation.

Our study has three main limitations. First, we did not manipulate the partner’s characteristics and the partner was always an adult. Since children typically look up to adults as role models and because they are obviously physically superior to children, there was not an equal relationship between partner and child. Additionally, because the adult partner was always in the room during the experiment, there was no opportunity for the children to tattle without being noticed by their partner. Thus, this setting might inhibit children’s active cheating or tattling because they fear revenge from the adult players^29^. Therefore, future work will examine children’s negative aspects of cooperation based on the different ages or attributes of their collaborative partners, such as peers^30^.

Second, we failed to adequately address developmental changes in terms of the negative aspects of cooperation. Although 3-year-olds’ tattling behavior did not reflect the degree to which the child and the partner were collaborating^15^, our study revealed that 7-year-olds changed their behavior. What developmental changes occur from ages 3 to 6 must also be examined. Berndt^31^ reported children tend to engage in more anti-social behavior as they grow, peaking around age 15^31^. Thus, examining developmental changes after children reach the age of seven may be fruitful. Furthermore, adults exhibited altruistic cheating even when the reward to the participant was fixed at a low level, even though the degree of this occurrence was low^7^. The processes underlying the development of altruistic cheating and its relationship with cognitive ability are additional future research topics.

Lastly, the effects of children’s background are in need of examination. First, previous studies revealed differences in the evaluations of blue lies between Asian and Western cultures. In detail, unlike Chinese children, Canadian children have been found to evaluate lies for self-interest more positively than lies for group-interest^32^. This difference may be derived from group-oriented Chinese cultural values, whereas Canadian culture is more individually-oriented. Considering these results, Western children might engage in fewer negative aspects of cooperation relative to Asian children. Thus, the effect of cultural values on the negative aspects of cooperation is an area ripe for future research. Second, it is important to examine the effect of SES on negative aspects of cooperation in children since previous studies have reported that SES affects children’s altruistic behavior, although these results reveal a trend that is inconsistent across studies^33–35^.

In sum, this study provided evidence that collaboration promotes the engagement of 7-year-olds in negative aspects of cooperation. Importantly, they engaged in negative aspects of collaboration not by actively creating them but by accepting the violations of others. Children also engaged in altruistic cheating for their partner, particularly when their reward was secured at a high level. Our findings suggest that, although 7-year-olds tend to believe that cooperating is more important than following rules when both are compatible, they behave strategically depending on the situation at hand. Although many studies reveal the positive aspects of cooperation in children, our study provides a novel viewpoint regarding the negative aspects of cooperation and its development.

## Methods

### Participants

Participants were recruited from children registered in our laboratory database, which was constructed by delivering flyers and through the lab website. Thirty-two 6- to 7-year-old children (M = 6 y 11 m, range: 6 y 6 m–7 y 4 m, N_girls_ = 16) participated in Experiment 1. Three children were excluded from analysis because they failed to comprehend the quiz game (2) or did not complete the task (1). Children were randomly divided into two conditions: individual and collaborative. An additional thirty-two 6- to 7-year-olds (M = 7 y 2 m, range: 6 y 7 m–7 y 7 m, N_girls_ = 16), who did not participate in Experiment 1 participated in Experiment 2 and were randomly divided into child-high and child-low conditions. After explaining our study’s content and methods, we obtained written informed consent from the parents of all participants. Participants received a small monetary remuneration for their participation. In addition, for publication of identifying images in an online open-access publication, we obtained informed consent from an adult player and the parents of the child participant in Fig. 1. The experiment was approved by the Ethics Review Board of the NTT Communication Science Laboratory. Experiments were carried out in accordance with the Code of Ethics and Conduct of the Japanese Psychological Association.

## Experiment 1

### Design

A child and another adult player earned coins based on correct answers and could exchange them for stickers based upon the following rules:

Individual condition: a child and another adult player individually gathered coins and earned rewards depending on the amount of earned coins.

Collaborative condition: a child and another adult player gathered coins as a team and shared the obtained rewards depending upon the number of coins.

In both conditions, one coin could be exchanged for two stickers.

### Procedure

A child, an adult player, and an experimenter participated in the experiment. The adult player was a research assistant who was meeting the children for the first time and was blind to the aim of the experiment. There were 7 adult players (N_male_ = 1, N_female_ = 6), and one person out of 7 people participated in experiment. We did not match the child and adult player based on gender since previous studies did not report the effects of combination of gender between target child and experimental assistant^16^. Before each experiment, adult partners were instructed not to teach quiz answers when the child was the respondent, but were instructed to puzzle the third question and finally cheat when they were the respondent. If a child talked to them during the task, they were asked to respond them naturally. Adults were instructed that it was not necessary to interact with the child voluntarily. After giving the child and the partner a chance to get to know each other for about 5 to 10 minutes, the experimenter began a modified version of the peeking paradigm^17^. In the peeking paradigm, a participant joined a quiz and was given an opportunity to cheat. We modified this paradigm to examine how children engage in the negative aspects of cooperation. In the modified design, two participants sequentially joined the quiz in a situation where they present each other’s question; that is, the non-answerer knows the answers (Fig. 1). The child and another adult player took turns participating in the quiz as the respondent and received a coin correct answers that could be traded for stickers.

In terms of the timeline of the task, the experimenter explained the rules of the quiz and how rewards are earned. The child and the adult player in the individual condition were told that they are individually participating in the quiz, whereas the children and the adult player in the collaborative condition were told that they are participating as a team. The experimenter re-confirmed that the children understood the relationship between the coins and the stickers. The child and the experimenter sat across a desk. As a respondent, the child turned his or her back to the experimenter, and as a non-respondent, the adult player observed both the child and experimenter (Fig. 1).

During the quiz, the child listened to animal sounds, such as barks or meows, and identified the animals only by listening to the sounds. The experimenter controlled a laptop PC and played the animal sounds from a speaker that was camouflaged as a puppet. The partner could see the answer (i.e., puppets). After answering, the child checked the answer by turning around. The experiment asked three questions per person, and guessing the correct answers of the first two was considered easy (e.g., dog, frog). However, the only way the child could correctly answer the last question was by turning around and cheating because the combination of puppet and sound (electronic sound) was arbitrary and guessing the correct answer from the sound was impossible. Before the third question, the experimenter’s mobile phone rang. The experimenter told the child that she had to leave the room for a minute and asked the child to delay his/her answer until she returned. After presenting the third question, the experimenter asked the adult player to make sure that the child didn’t cheat and left the room. After about a minute, the experimenter returned and asked for the child’s answer. After the child’s turn, the adult player’s turn was conducted in the same way except for the following two changes. First, the adult player cheated on the last question by turning around after one minute from the time when the experimenter left the room and asked the child not to disclose the cheating. Second, the experimenter returned after about 90 seconds. Importantly, there was no opportunity for the children to tattle without being noticed by their partners. After the player’s turn, the child and the adult player were given the opportunity to exchange their coins for stickers and they were debriefed that the adult player was asked to cheat in advance. Although the experimenter stated that cheating by the adult partner is bad behavior, the experimenter affirmed the child’s behavior regardless of whatever action they took.

### Coding

To examine the negative aspects of cooperation in children, we coded three types of behavior: (a) whether the child cheated when they were thinking about the answer to the third question when the experimenter was absent; (b) whether the child provided hints about the adult player’s third question when the experimenter was absent; and (c) whether the child tattled to the experimenter when the adult player cheated. Furthermore, the target duration of the analysis of the children’s tattling ranged from when the experimenter re-entered the room to when the coins were exchanged for stickers. Behaviors (a) and (b) denote actively cheating, whereas behavior (c) denotes passively cheating.

## Experiment 2

### Design

Child-high condition: a child and an adult player gathered coins as a team. While the adult player’s reward was determined by the sum of coins earned by the team, the child’s reward was predefined as six stickers regardless of the number of coins.

Child-low condition: although a child and an adult player earned coins as a team, the adult player’s reward was determined by the amount of coins gained by them together. The child’s reward was predefined as one sticker regardless of the number of coins.

In both conditions, the adult player can exchange a coin for a sticker. That is, the child’s reward did not fall below the adult player’s reward in the child-high condition, while the child’s reward did not exceed the adult player’s reward in the child-low condition.

### Procedures

Except for a change in the amount of the child’s predefined reward, the procedure in Experiment 2 was identical to the collaborative condition in Experiment 1.

### Coding

The behavior indexes were identical to Experiment 1.

### Data availability

All data generated or analyzed during this study are included in this published article.
